# 

**Published:** 1835-01-01

**Authors:** 


### CONTENTS

OF THE

### MEDICO-CHIRURGICAL REVIEW,

### No. XLIII. JANUARY 1, 1835.

[BEING No. III. OF A DECENNIAL SERIES.]

### REVIEWS.

I.

Dr. and Mr. GRIFFIN on Functional Affections of the Spinal Cord and Ganglionic Nerves .. .. .. .. .. .. .. .. .. .. .. .. .. .. .. .. .. 1

II.

Dr. COXE's Inquiry into the Claims of Dr. HARVEY to the Discovery of the Circulation 31

III.

M. [illegible]YTREN and Mad. BOIVIN on Fibrous Tumors and Polypi of the Uterus .. .. 34

IV.

Experiments and Observations on the Gastric Juice, and the Physiology of Digestion. By Dr. BEAUMONT .. .. .. .. .. .. .. .. .. .. .. .. .. .. .. .. .. 49

V.

Memoirs of the Life and Medical Opinions of Dr. Armstrong. By Dr. BOOTT. [Malaria and Marsh Fever.] .. .. .. .. .. .. .. .. .. .. .. .. .. .. .. 69

VI.

The Principles and Practice of Obstetricy. By Dr. BLUNDELL .. .. .. .. .. .. 73

VII.

The Concours—Clinical Chair of Surgery, Paris.

1. Parallele entre La Taille, &c. BLANDIN .. .. .. .. .. .. .. .. } 79

2. Des Hæmorrhoides, &c. LEPELLETIER .. .. .. .. .. .. .. .. } 79

3. Obliteration des Arteres. LISFRANC .. .. .. .. .. .. .. .. } 79

VIII.

Mr. SCOTT on Tic Douloureux and Neuralgia .. .. .. .. .. .. .. .. .. .. .. 97

IX.

Mr. GUTHRIE on the Anatomy and Diseases of the Neck of the Bladder, &c. .. .. 107

X.

Dr. QUAIN's Elements of Anatomy .. .. .. .. .. .. .. .. .. .. .. .. .. .. 137

XI.

Mr. WARDROP's Morbid Anatomy of the Human Eye .. .. .. .. .. .. .. .. .. 140

XII.

Mr. MAYO's Introductory Lecture to the Medical Classes in King's College, London .. 141

### PERISCOPE.

### PART I.

### Spirit of the English Periodicals, and Notices of English Medical Literature.

1. Practical Hints on the Treatment of several Diseases. By Dr. Peacock .. .. 145
    1. Extensive Sloughing—2. Gangrene—3. Diabetes.—4. Dropsy—5. Neuralgia —6. Pain from Exhaustion—7. Anomalous Cases—8. Obstinate Constipation.
2. Mr. Latham on Uterine Hæmorrhage.. .. .. .. .. .. .. .. .. .. .. .. .. 150
3. Dr. De Mey on the Triumph of Truth and good Sense .. .. .. .. .. .. . 150
4. Dr. Leese's Treatise on Gout .. .. .. .. .. .. .. .. .. .. .. .. .. .. 151
5. Dr. Roberts on Hemiplegia in Pregnancy .. .. .. .. .. .. .. .. .. .. .. 152
6. Dr. Stroud's Case of Carditis—or Cephalitis .. .. .. .. .. .. .. .. .. 153
7. Endemic Dysentery in the Edinburgh Workhouse .. .. . .. .. .. .. .. 154
8. Pseudo-pneumonia—Dr. Hamilton on .. . .. .. .. .. .. .. .. .. .. 155
9. Dr. Hawthorne on Cholera.. .. .. .. .. .. .. .. .. .. .. .. .. .. .. 156
10. Professor Beatty on "Frottement" observed in Peritonitis .. .. .. .. .. .. 160
11. Sir Charles Bell—the mighty Dead .. .. .. .. .. .. .. .. .. .. .. .. 161
12. Dr. M'Dowel on Erysipelas .. .. .. .. .. .. .. .. .. .. .. .. .. .. 162
13. On Gastric Neuralgia. By the Senior Editor .. . .. .. .. .. .. .. .. 166
14. Crepitatio Musculorum. By Dr. Johnson .. .. .. .. .. .. .. .. .. .. 168
15. Plurality of Assistants in Hospitals .. .. . .. .. .. .. .. .. .. .. 168
16. Anatomico-pathological Researches on the Pneumogastric Nerves .. .. .. .. 170
17. Mr. Poole on the Pathology of Tubercular Phthisis .. .. .. .. .. .. .. .. 171
18. Phlegmasia Cærulea Dolens .. .. .. .. . .. .. .. .. .. .. .. .. .. 173
19. Dr. Montgomery on Transverse Malposition .. .. .. .. .. .. .. .. .. .. 173
20. Incipient Phthisis cured—Dr. Forsyth .. .. .. .. .. .. . .. .. 177
21. Dr. Parrish on Ununited Fracture cured by Pressure .. .. .. .. .. .. .. 177
22. Dr. Huston on Hypertrophy of the Mammæ .. .. .. .. .. .. .. .. .. .. 179
23. Mr. Pettigrew's Clinical Lecture on Hydrophobia .. .. .. .. .. .. .. .. 180
24. Dr. Thorburne's Proposal to dislocate the Head of the Femur in Morbus Coxarius, 183
25. Dr. Campbell's Case of Pendulous Tumors growing from the Ear .. .. .. .. 186
26. Occlusion of the Ductus Communis Choledochus .. .. .. .. .. .. .. .. .. 187
27. Prevalence of Calculous Complaints in India .. .. .. .. .. .. .. .. .. 187
28. Dr. Edwards on the different Races of Mankind .. .. .. .. .. .. .. .. 189

### PART II.

### Spirit of the Foreign Periodicals.

9. On the Employment of large Doses of Antimony after severe Wounds, &c. .. .. 193
30. Wretched State of Midwifery in Germany .. .. .. .. .. .. .. .. .. .. .. 193
31. The Virtues of Sarsaparilla appreciated in Germany. .. .. .. .. .. .. .. 194
32. Character of Continental Medical Literature .. .. .. .. .. .. . .. 194
33. Lithotomy in Italy .. .. .. .. .. .. .. .. .. .. .. .. .. .. .. .. 196
34. Operations of Lithotomy and Lithotrity—in illustration of M. Blandin's Memoir, reviewed in this number .. .. .. .. .. .. .. .. .. .. .. .. .. 197
35. On Hæmorrhoids and Prolapsus of the Rectum.. .. .. .. .. .. .. .. .. 204
36. On Cutaneous Diseases. By M. Gibert .. .. .. .. .. .. .. .. .. .. .. 206
37. Endermic Use of Digitalis in Dropsy .. .. .. .. .. .. .. .. .. .. .. 207
38. Strictures on the Broussaian Method of investigating Diseases.. .. .. .. .. 209
39. New Preparation of Quinine—Hydro-ferro-cyanate .. .. .. .. .. .. .. .. 211
40. Varicose Tumor on the Scalp .. .. .. .. .. .. .. .. .. .. .. .. .. 212

41. Blue Serum from a blistered Surface .. .. .. .. .. .. .. .. .. .. .. .. 213
42. Anecdote of Vesalius .. .. .. .. .. .. .. .. .. .. .. .. .. .. .. 213
43. Ligature of the Subclavian Artery. Dr. Sentin .. .. .. .. .. .. .. .. 214
44. On Fractures of the Carpal Extremity of the Radius .. .. .. .. .. .. .. .. 216
45. Pathological Anatomy of the Pneumo-gastric Nerves .. .. .. .. .. .. .. 218
46. Anatomy of the Lymphatic System .. .. .. .. .. .. .. .. .. .. .. 220
47. Melanosis of the Eye-ball .. .. .. .. .. .. .. .. .. .. .. .. .. 221
48. Phrenology, human and comparative .. .. .. .. .. .. .. .. .. .. .. 221
49. Detection of Miasmata in the Air. M. Bousingault .. .. .. .. .. .. .. 223
50. Rupture of Varices of the Vagina during Labour .. .. .. .. .. .. .. 224
51. Dr. Bellingheri on Facial Neuralgia .. .. .. .. .. .. .. .. .. .. .. 225
52. Professor Albers on Inflammation of the Spinal Dura Mater .. .. .. .. .. .. 227
53. Singular Case of Epileptic Hysteria .. .. .. .. .. .. .. .. .. .. .. 229
54. Neuralgia of the Leg and Foot—Excision of Nerve .. .. .. .. .. .. .. 229
55. Two Cases of Successful Transfusion of Blood in Hæmorrhage .. .. .. .. .. 230
56. Bouillaud's Refutation of Majendie's Theory of the Heart's Sound .. .. .. .. .. 231
57. Secale Cornutum as an Emmenagogue .. .. .. .. .. .. .. .. .. .. .. 233

PART III.

### Clinical Review and Hospital Reports.

St. George's Hospital.

58. Abstract of a Lecture on Injuries of the Nerves, delivered by Cæsar Hawkins, Esq. 234

Charing-Cross Hospital.

59. Surgical Report.
A. Fractured Ribs—Laceration of the Hand and Arm .. .. .. .. .. .. .. 241
B. Fractured Ribs .. .. .. .. .. .. .. .. .. .. .. .. .. .. .. .. 241
C. Fractured Femur—no Union—Death .. .. .. .. .. .. .. .. .. .. .. 242
D. Compound Fracture of the Tibia and Fibula—Death .. .. .. .. .. .. .. 243
E. Removal of a Steatomatous Tumor .. .. .. .. .. .. .. .. .. .. .. 243

St. Thomas's Hospital.

60. Dr. Roots' Case of Neuralgia .. .. .. .. .. .. .. .. .. .. .. .. 244

Manchester Royal Infirmary.

61. A. Chorea .. .. .. .. .. .. .. .. .. .. .. .. .. .. .. .. .. 246

B. Jaundice .. .. .. .. .. .. .. .. .. .. .. .. .. .. .. .. .. 248

C. Rheumatism .. .. .. .. .. .. .. .. .. .. .. .. .. .. .. .. 251

D. Amenorrhœa .. .. .. .. .. .. .. .. .. .. .. .. .. .. .. .. 254

New Westminster Hospital.

62. Mr. Guthrie's Clinical Lecture .. .. .. .. .. .. .. .. .. .. .. .. 256

Pennsylvania Hospital.

63. A. Injuries of the Head .. .. .. .. .. .. .. .. .. .. .. .. .. .. 259
B. Case of Phlebitis after Operation for Varicose Veins .. .. .. .. .. .. .. 264

St. Thomas's Hospital.—*(bis.)*

64. On the Treatment of Syphilis without Mercury .. .. .. .. .. .. .. .. 264

### Miscellanies.

65. Case of Hepatized Spleen. By James Edward, Surgeon, of Forfar .. .. .. 272

66. Medical Education and Organization .. .. .. .. .. .. .. .. .. .. .. 272

Bibliographical Record .. .. .. .. .. .. .. .. .. .. .. .. .. .. .. 278

### EXTRA-LIMITES.

The Baths of Pfeffers in the Grison Country. By JAMES JOHNSON, M.D. .. .. 281
Singular romantic situation of the Bath-House .. .. .. .. .. .. .. .. .. 282
Gorge of the Tamina—frightful cavern.. .. .. .. .. .. .. .. .. .. .. 284
Source of the Thermal Waters in the Cavern .. .. .. .. .. .. .. .. .. 286
A German Table D'Hôte.. . .. .. .. .. .. .. .. .. .. .. .. .. .. .. .. 291
The Waters of Pfeffers, and the singular Mode of using them .. .. .. .. .. 296
Remarks on Thermal Waters and Warm Bathing in various Complaints .. .. 300

